# What Affects an Orthopaedic Surgeon's Online Rating? A Large-Scale, Retrospective Analysis

**DOI:** 10.5435/JAAOSGlobal-D-22-00027

**Published:** 2022-03-15

**Authors:** Mital D. Patel, Marshall D. Williams, Merritt J. Thompson, Parth N. Desai

**Affiliations:** From the Mercer University School of Medicine, Macon, GA (Mr. Patel, Mr. Williams, and Mr. Thompson), and the Ochsner Health, Department of Orthopedic Surgery, New Orleans, LA (Dr. Desai).

## Abstract

**Introduction:**

In the past decade, online physician review websites have become an important source of information for patients, with the largest and most popular being Healthgrades.com. Our study aims to investigate demographic and volume-based trends for online reviews of every Healthgrades-listed orthopaedic surgeon through a nationwide, retrospective analysis.

**Methods:**

All available demographic and rating information for orthopaedic surgeons (n = 28,713; Healthgrades.com) was analyzed using one-way Analysis of Variance, Tukey Studentized Range (Honestly Significant Difference), linear regression, and Pearson correlation coefficient.

**Results:**

The mean rating for all surgeons was 3.99 (SD 0.92), and the mean number of ratings was 13.43 (SD 20.4). Men had a greater mean rating at 4.02 compared with women at 3.91 (*P* < 0.0001), and DO surgeons had greater mean rating at 4.11 compared with MD surgeons at 3.90 (*P* < 0.0001). The correlation between rating and age had a significant negative correlation (*P* < 0.0001). The correlation between average online rating and number of reviews had a significant positive correlation (*P* < 0.0001).

**Discussion:**

Our analysis suggests that greater online ratings are associated with the male sex and DO degrees. In addition, our study discovered that the number of ratings was positively correlated with greater mean online ratings, whereas older age was negatively correlated with greater mean online ratings.

With the advent of digital information technology, online physician review websites have become a major source of information for patients. Studies suggest that 25% to 72% of Americans use online reviews when selecting a healthcare provider.^1,2^ As of 2010, over 33 different physician rating websites had been identified,^3^ with the largest and most popular being Healthgrades.com.^4^ Healthgrades.com allows anyone with internet access to rate a physician or hospital from one to five stars in several different categories. The mean of all reviews is posted anonymously on the physician's public profile along with optional comments from reviewers. For most physicians, the first Google search results of the physician's name include their online rating from different physician review sites. These ratings can affect practice growth, patient acquisition, employment opportunities, malpractice litigation, and overall reputation. Given the implications of these reviews, this rating system is becoming a major issue among physicians, with one study reporting that as many as 69% of physicians have checked their online review profiles at least once.^5^ As physician reviews expand in importance, so too does the controversy surrounding them.

Critics cite a number of harmful consequences related to the growth of online physician reviews. Online reviews of physicians are subject to a great degree of voluntary response bias. One study of a large academic orthopaedic practice found that only 2% of the respondents had left an online review.^6^ Another study found that the most common reviewers were younger patients, female patients, highly educated patients, and those with greater healthcare utilization.^7^ Although the literature is sparse, it is widely thought that disgruntled patients are more likely to post negative reviews, whereas satisfied patients are less likely to leave any reviews. A physician's total rating can potentially be volatile and subject to sampling bias, especially if there are a limited number of total reviews used to calculate their overall rating. In addition, a review website has no way of verifying whether a reviewer is truly a patient; thus, fraudulent reviews can be left to damage a physician's reputation. Competing practices or disgruntled employees can also leave negative reviews to discredit a physician. As such, reviews may not be accurate indicators of the quality of care provided by a clinician. Several studies have reported that hospital rankings on Healthgrades.com are not accurate indicators of patient care outcomes and provide inadequate assessments of a physician.^8,9,10,11^

In addition, the argument can be made that physicians seeking to increase their online rating by soliciting reviews from satisfied patients and those with positive outcomes is an ethical violation. The American Psychological Association Ethics Code, principle 5.05 states that “Psychologists do not solicit testimonials from current therapy clients/patients or other persons who because of their particular circumstances are vulnerable to undue influence.^12^” Orthopaedic patients are similarly vulnerable to and under undue influence. Therefore, physicians are placed in a difficult position of facing the detrimental effects of negative reviews while also falling into an ethically questionable territory when trying to improve their scores. The combination of these factors has led to reports of emotional and psychological stress for physicians induced by online reviews.^13^

Conversely, proponents of online physician reviews state that these websites improve transparency by giving patients a voice to express their healthcare experiences. These sites may also improve patient autonomy by empowering patients to make a more informed decision when selecting a healthcare provider^14^ and benefit physicians looking to improve the quality of their care.^15^ One apparent benefit is that many of these websites provide other useful information for patients such as a physician's office address, office hours, phone number, sex, age, education, and board certifications. Although it is uncertain whether physician reviews are harmful or beneficial, it seems that they are here to stay.

Due in part to the large number of elective surgical procedures, orthopaedic surgeons represent a key specialty affected by online physician reviews. Overall, there is a general paucity of literature analyzing trends for orthopaedic surgeon reviews.^14,16,17,18,19,20,21^ The few previous studies of orthopaedic surgeons have only been able to analyze small subsets of reviews because of the manual process of collecting online review data. To date, no previous study has been able to gather the entire data set of every orthopaedic surgeon in the United States listed on Healthgrades.com. Thus, our study intends to be the first large-scale demographic and trends analysis of all orthopaedic surgeons in the Healthgrades.com database.

## Methods

A tool for automated extraction of publicly available Healthgrades.com data was developed in the programming language Python. On April 15, 2018, the program compiled the demographic and rating information for every registered orthopaedic surgeon on the Healthgrades.com open-access website. Standard accepted protocols for web scraping and automation were followed.^22^ For each listed orthopaedic surgeon, the name, age, sex, address, mean online rating, and number of patient reviews were gathered and exported in .csv format. Only MD and DO orthopaedic surgeons were included in this study, with all other healthcare providers excluded, leading to a total of 28,713 physicians. One-way Analysis of Variance (ANOVA) and Tukey Studentized Range were used to analyze the effects of the demographics on mean online rating, specifically comparing the rating differences between sex, the MD versus DO degrees, and age. Linear regression and Pearson correlation coefficient were used to analyze the relationship between the average online rating and the number of reviews and also between age and mean online rating. Statistical significance was defined as *P* < 0.05. All statistical analysis was performed using SPSS version 25.0 (IBM).

## Results

Of the 28,713 orthopaedic surgeons included in the analysis, the mean age of surgeons was 58.3 years (SD 15.2), with 94.7% men to 5.3% women and 93.5% MD surgeons to 6.5% DO surgeons. The mean rating for all surgeons was 3.99 (SD 0.92), and the mean number of ratings was 13.43 (SD 20.4). The difference in total mean ratings was statistically significant based on sex and medical degree. Men had a greater mean rating at 4.02 (SD 0.91) compared with women at 3.91 (SD 0.95) with a *P* value of <0.0001. DO surgeons had greater mean rating at 4.11 (SD 0.82) compared with MD surgeons at 3.90 (SD 0.92) with a *P* value of <0.0001. Rating and age had a significant negative correlation, R = −0.312, with a *P* value of <0.0001. Linear regression between rating and age resulted in a slope coefficient of −0.02, a slope standard error of 0.0019, and a *P* value of <0.0001. Average online rating and number of reviews had a significant positive correlation, R = 0.23, with a *P* value of <0.0001. Linear regression between average online rating and number of reviews resulted in a slope coefficient of 0.015, a slope standard error of 0.0024, and a *P* value of <0.0001.

## Discussion

Online physician rating websites are becoming an increasingly important source of information for patients looking to establish care. These websites provide a unique platform in which patients can review their experiences with a physician, read other patients' reviews, form opinions, and gain further insight into how a potential provider may match with a patient. Orthopaedic surgery in particular is a field saturated in medical consumerism where many procedures are elective in nature. Physician rating websites are a valuable resource where patients can look for providers specializing in the specific care they desire and find subjective information that may influence their decision to pursue care.^23^ For online patient ratings on the Healthgrades.com platform, the criteria in physician evaluations can be categorized as trustworthiness, explains condition(s) well, answers questions, and time well spent. Numerous other studies have evaluated online physician rating websites and social media usage, specifically in the field of orthopaedic surgery.^16,17,24,25^ However, our study is the first to analyze such a broad scope of physician ratings, transcending individual subspecialties to include every single orthopaedic surgeon on Healthgrades.com as of 2018. The results of our analysis demonstrate that a higher total number of ratings positively correlate with higher mean online ratings. In addition, male sex and DO medical degree are associated with greater online ratings after accounting for the effect of volume on mean online ratings. Although the numerical difference in our findings is slight (4.02 male versus 3.91 female and 4.11 DO versus 3.90 MD) and may not reflect surgical skill or patient outcomes, they will affect a practice. We will look further into these differences because the significance lies in how the public uses these grades to guide their choice of surgeon. Those with the highest grades will draw a larger patient volume and, by extension, reap more financial benefits.

Several studies have analyzed the correlation between physician ratings with physician sex. A number of studies have found no correlation between the two variables^21,26,27^; however, others have found mixed association.^10,28,29^ The surgical fields in general have historically been male-saturated, and orthopaedic surgery is certainly no exception. In our study, the extracted data from Healthgrades.com divided the orthopaedic surgeon pool into 94.7% men and 5.3% women, with a significantly higher rating among men versus women (4.0 versus. 3.9 [*P* < 0.0001]). Positive doctor-patient relationships are a strong determinant in patient satisfaction and clinical outcome, with empathy being particularly important to skilled communication.^30^ A study by Chaitoff et al^31^ found that female physicians have higher empathy scores, while a study by McTighe et al^32^ demonstrated higher empathy scores among female medical students. Roter et al^27^ demonstrated that female providers participate more actively than their male counterparts in a number of categories fundamental to the physician-patient relationship, including, but not limited to, positive talk, psychosocial counseling, and higher interaction time. These findings suggest that higher male provider scores compared with female provider scores may very well be counterintuitive when analyzing specific aspects of patient care. Additional research could be made comparing the differences found between men and women in the categories listed on Healthgrades: trustworthiness, explains condition(s) well, answers questions, and time well spent. This analysis would offer helpful insight into particular areas that physicians could focus on to increase their scores.

The markedly higher ratings of DO surgeons is one of the most intriguing findings. One factor regarding the difference may lay in aspects of DO training that are not as heavily emphasized in the MD counterpart. Osteopathic medical schools differentiate themselves from their allopathic contemporaries through the core principles of the biopsychosocial model and a greater emphasis on the interconnectedness of the mind and body.^32^ Theoretically, the empathetic nature of a medical student should continue through to their quality of care as a practicing physician after completion of medical school.^33^ This respect for and application of their core principles in training could lead to greater empathy in practice. Studies on the general relationship between medical education and the development and retainment of empathy reported that medical education is associated with a decrease in empathy as students progress to later years.^34,35^ Studies specific to osteopathic medical education, however, did not find a notable decrease in empathy levels like in allopathic medical education; although on graduation, there was no notable difference in empathy between the two training methods.^36,37^ This potential connection between osteopathic education and increased empathy as a medical practitioner seems logical, although there remains more speculation than certainty at this time. This speculation on the greater degree of empathy in osteopathic graduates and the potential relationship between empathy, patient care, and online ratings could contribute to the explanation of our findings of markedly higher orthopaedic physician ratings in osteopathic doctors.

A study by Donally et al^17^ analyzed variation of ratings between DO versus MD spine surgeons among Healthgrades.com, Vitals.com, and Google.com but found no notable difference in the ratings between the degrees. An important consideration is that we only sampled from Healthgrades.com, while Donally et al sampled from three major websites. However, the method of sampling contrasted between their study and ours. The difference in results could be attributed to the sheer difference in sample size. Their study analyzed the subset of orthopaedic spine surgeons (n = 206), while we included all orthopaedic surgeons, regardless of specialty (n = 28,713). Regardless of the medical degree, it may behoove orthopaedic surgeons looking to improve their online reviews to conduct a patient-centered interview with an emphasis on empathetic interactions.

In our study, a greater total amount of ratings were strongly positively correlated with greater mean online ratings. Other studies have shown that social media involvement, even just updating and maintaining a professional profile on the review site in question, resulted in a greater number of reviews not only for an individual orthopaedic surgeon but also for an orthopaedic group as a whole.^16,24^ The higher number of reviews aligning with higher ratings may be a direct consequence of physicians encouraging satisfied patients to complete reviews. Encouraging the completion of reviews is becoming a common practice in most fields with a growing online presence. Although the use of social media has been questioned as an acceptable mode of communication for physicians, it has also been shown to be an effective method to convey education, share knowledge, and improve health outcomes.^25^ With more physicians using social media as a way of communication, future guidelines will need to be constructed to preserve patient confidentiality and physician liability.^38^

Similar to the study by Imbergamo et al,^39^ our analysis demonstrates that rating and age have a significant negative correlation (*P* < 0.0001), demonstrating that younger physicians often obtain higher ratings. Although patient feedback through online rating services may not remain positive or strictly correlate with physician competency,^20^ this additional feedback may be beneficial nevertheless. Physicians in the best position to take advantage of this opportunity seem to be those who have recently graduated or established a practice. Younger physicians have been shown to value a presence on social media and think it can be used to benefit their career.^40^ This belief combined with a proficient use of newer technologies positions these physicians to use online ratings to their advantage to bolster their patient load and grow their clinical practice.

Care should be taken that an increased social media presence is not the only change made by a modern orthopaedic surgeon. Although multiple studies show that social media presence for surgeons results in an increased engagement with patients on review platforms and a corresponding higher total amount of online reviews,^16,24^ there is a caveat in this group that does not follow expected trends. Contrary to our findings, in these studies more reviews did not necessarily correlate with better ones. The overall average review scores for surgeons with a social media presence were identical to those without one, despite receiving more feedback.^16,24^ These same studies showed a positive correlation between shorter wait times and higher scores for patients. Other identified areas for improvement included concrete factors such as physician punctuality, the ease with which a patient can schedule an appointment, positive interactions with staff, and personal perceptions of physician competence and trustworthiness.^19^

These findings imply that social media presence alone is just a single factor in improving a physician's online perception. Additional measures are necessary for a positive online presence, be it improving the experience at the clinic, using social media as a platform for patient communication, or the increased focus on empathy and interpersonal skills discussed earlier. Using these data, we recommend that orthopaedic surgeons interested in improving their mean online rating should implement strategies that focus on increasing their volume of patient reviews, such as requesting that all patients leave an honest online review. Social media use and proficiency may be a future indicator of online review ratings because having an online presence allows physicians to connect with their patients in a way unavailable to their colleagues who do not have an established online presence.^17^ To expand the scope of future studies, patient review data should be obtained from other major online physician review sites.

There are some limitations that must be addressed concerning our research. First, many studies face limitations demonstrating sex differences. These limitations include small sample sizes and stratification of sex in specific medical specialties (94.7% men and 5.3% women in orthopaedics). Implicit bias in a historically male field could perpetuate and contribute to disproportionate ratings. As medicine becomes more female-populated, this type of bias could resolve over time. Second, although our study looked at the mean rating for all surgeons (3.99) and the mean number of ratings (13.43), we did not extrapolate to find the total reviews for MDs and DOs or the mean number of ratings for MDs and DOs. This opens the possibility that our notable difference could be biased based on the skewed representation between MDs and DOs in the field, similar to how sex is skewed in representation.
